
JOURNAL INFORMATION
==============================
NLM Title Abbreviation: ScientificWorldJournal
Journal ID: ScientificWorldJournal
NLM Journal ID: 101131163
Journal ID: TSWJ

The Scientific World Journal

ISSN: 2356-6140
EISSN: 1537-744X
Publisher: Wiley

ARTICLE INFORMATION
==============================
PMCID: PMC5956306
PMID: 15349551
DOI: 10.1100/tsw.2004.76
Article ID: 735051
Article version: 1
Subjects: Research Article

Diagnosis, Evaluation and Treatment of Adolescent Varicocele

Paduch Darius A.
Skoog Steven J.
Division of Urology and Renal Transplantation, Oregon Health Sciences, University, Portland, OR, USA
Academic Editor: Anthony Atala

Publication date: 2004
Electronic publication date: 2004 Jun 7
Volume: 4
First page: 263
Last page: 278
Copyright: Copyright © 2004 Darius A. Paduch and Steven J. Skoog.
Copyright year: 2004
License: This is an open access article distributed under the Creative Commons Attribution License, which permits unrestricted use, distribution, and reproduction in any medium, provided the original work is properly cited.
License URL: https://creativecommons.org/licenses/by/3.0/

==============================
Articles
*TheScientificWorldJOURNAL* (2004) 4 (S1), 263–278
ISSN 1537-744X; DOI 10.1100/tsw.2004.76

### Diagnosis, Evaluation and Treatment of Adolescent Varicocele

Darius A. Paduch and Steven J. Skoog
*Division of Urology and Renal Transplantation, Oregon Health Sciences University, Portland, OR*

**E-mails**: paduchd@ohsu.edu

*Previously published in the Digital Urology Journal*

---

**DOMAIN**: urology

---

### INTRODUCTION

A varicocele can be defined as an abnormal tortuosity and dilation of the veins of the pampiniform plexus. Idiopathic varicocele is usually asymptomatic. It is noticed as an asymmetry in scrotal size, and presents as heaviness in the scrotum, or rarely with testicular pain. In most cases the adolescent is unaware of the varicocele and it is discovered during a regular physical examination or during examination for military service.[1-4]

The incidence of high-grade varicocele is approximately 5 % throughout the world.[5] Varicocele is associated with a time dependant growth arrest in adolescents and adult males.[6] There is a clear association between varicocele, infertility and testicular growth arrest.[7-9] It is also known that varicocelectomy can reverse testicular growth arrest in adolescents.[10-13] These facts have raised the question of how to best manage the adolescents with a varicocele.

Adolescents do not present with infertility and thus should prophylactic repair be performed to prevent infertility in the future? Who would benefit the most by varicocelectomy: the adolescents with testicular growth arrest or any adolescent with a varicocele? Is it better to wait for a semen analysis or offer earlier treatment based on testicular growth arrests? These questions can be only answered when we have better understanding of the pathophysiology of varicocele.

The purpose of this review is to present the current literature on adolescent varicocele and provide guidelines to the clinician how to manage the adolescents with a varicocele.

### EPIDEMIOLOGY

In the general population of healthy males the overall incidence of varicocele (all grades) is 10% to 15%.[4,5,14,15] Approximately 30-50 % of males with primary infertility have a varicocele.[16-19] Varicocele is most common on the left side. Varicocele appears at early puberty however it can occasionally be found in preadolescent boys.[2,20] The incidence in older adolescence varies from 12.4 % to 17.8 % with an average of 14.7 %, similar to the incidence in adult males. (Table 1.)

**TABLE 1**
**Incidence of varicocele in general population of healthy adolescents.**

| Reference: | No of patients: | Age: | Incidence: (total) |
|---|---|---|---|
| Oster, 1971[101] | 837 | 10-19 | 16.2% |
| Steeno, 1976 [103] | 4067 | 12-25 | 14.7% |
| Yerokhin, 1979 [146] | 10000 | 10-17 | 12.4% |
| Belloli, 1993 [9] | 9861 | 10-16 | 16.0% |
| Niedzielski, 1997 [5] | 2478 | 10-20 | 17.8% |

### ANATOMY

The arterial blood supply to the testis comes from the testicular artery, vasal artery and the cremasteric artery. At the level of testis all three arteries anastomose to allow adequate blood supply even with division of the testicular artery.[21,22]

The venous drainage (Figure 1) is more complicated with many individual variations. Above the testis is a network of communicated veins called the pampiniform plexus (PP), the drainage from PP is via the testicular vein trunci, pudendal veins and cremasteric veins.[23,24] In most cases the testicular vein trunci form a single testicular vein entering the renal vein on the left and the inferior vena cava on the right. Venographic studies have demonstrated that the left testicular vein can rarely enter the inferior vena cava, and there are communications between the testicular vein and the inferior vena cava below the level of the renal veins.[25-27]

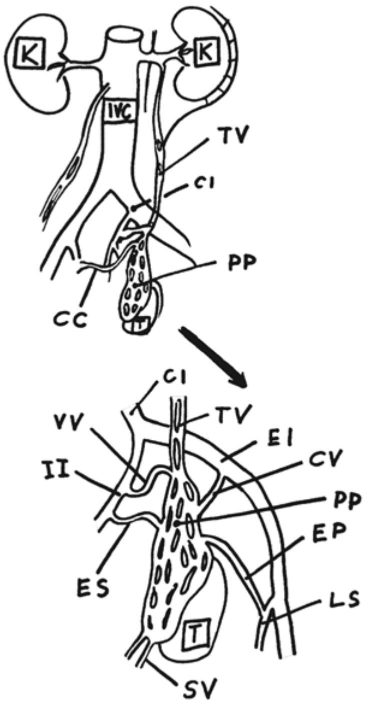

**FIGURE 1**. Anatomy of venous drainage from left testis.

There are also cross-communications between the left and right testicular venous systems (Figure 2).[26,28-30]

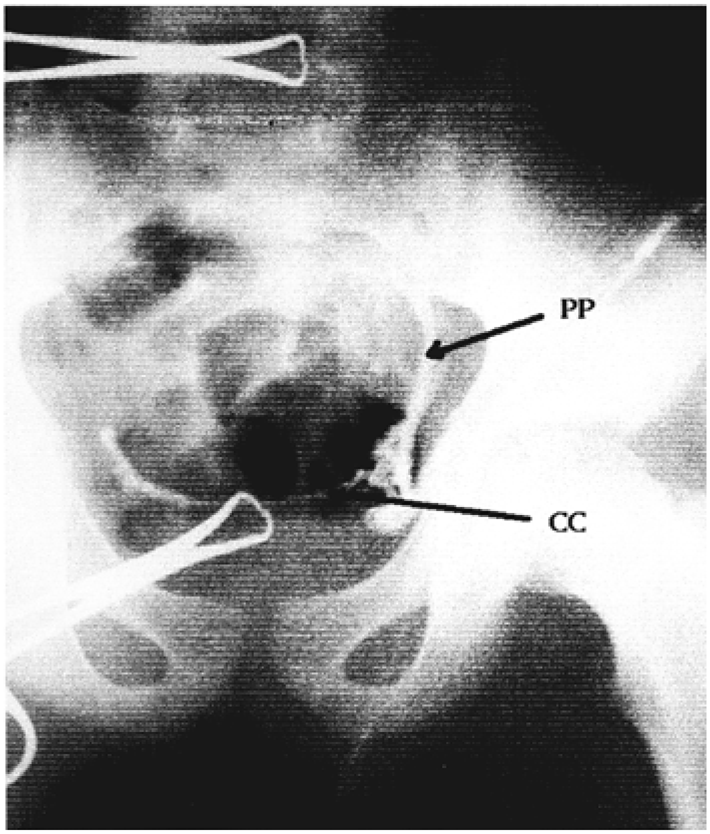

**FIGURE 2**. Intraoperative venogram showing left to right cross-communicating veins CC-cross-communications, PP-pampiniform plexus veins

### ETIOLOGY

There are several theories attempting to explain the etiology of varicocele. The predominance of the left side varicocele and the unique anatomy of the left testicular vein are the basis for several theories explaining the etiology of varicocele.

The presence of venous valves was long believed to be a guarding mechanism against developing a varicocele and incompetence of the venous valve system was thought to be responsible for varicocele development. However, it was shown that there are males without varicocele who have incompetent venous valve system and males with varicocele who had competent venous valves.[29]

Second, because the left testicular vein is longer than the right, the hydrostatic pressure difference could be a factor causing a left varicocele. Although the left testicular vein is longer than the right, the simple difference in hydrostatic pressure of a standing column of blood can not be the only reason for development of a varicocele because all males would be affected.

A third theory known as a "nutcracker effect" is speculated to occur when the testicular vein is compressed between the superior mesenteric artery and aorta. The increase in hydrostatic pressure results in varicocele formation. However although high left renal vein to vena cava pressure gradients are noted in patients with varicocele, it is not a consistent feature.[31,32]

More recently it has been hypothesized that increased arterial blood flow to the testis at puberty exceeds the venous capacity resulting in venous dilatation and a varicocele.[33,34] This is consistent with the findings noted in all animal models studied to date, however confirmation in humans will be necessary.

### PATHOPHYSIOLOGY

The pathophysiology of varicocele can be studied in animal models by partial ligation of the left renal vein.[35] Many features of the human condition, like increased temperature of the affected testis, increased arterial blood flow and histopathological changes can be replicated in animal models.

The following theories attempt to explain the deleterious effect of varicocele on testicular function.

### Hyperthermia

The presence of a varicocele is associated with elevated scrotal and testicular temperature and altered spermatogenesis. Experimental studies have shown that spermatogenesis occurs optimally at temperatures lower than body temperature. Many of the enzymes responsible for optimal DNA synthesis in the testis are temperature dependent.[36,37] The scrotal position of the testis and the cooling system provided by the pampiniform plexus surrounding the testicular artery allows for heat exchange and is responsible for regulating optimal temperature for spermatogenesis.[38] Stasis of blood in the varicocele with resultant increased temperature may be responsible for the deleterious effect of varicocele on spermatogenesis.[39] Increased temperature is associated with decreased number of spermatogonia and increased apoptosis of germinal epithelium cells.[40]

### Hypoxia and "adrenal reflux"

Stasis of blood in pampiniform plexus could affect partial oxygen pressure and change aerobic metabolism in the testis. However hypoxia has not been demonstrated in testicular venous blood sampling in humans or experimental animals.[41,42] Reflux of blood down the testicular vein has been demonstrated in patients with varicocele.[43,44] Therefore exposure of the testis to adrenal or renal metabolites is hypothesized as cause for testicular damage. However adrenal or renal metabolites at the level of the testis have not been documented.[35,45] Adrenalectomy done on rats with experimental varicocele did not diminish the effects of the varicocele.[46,47] Thus the adrenal/renal reflux theory does not appear to be responsible for the testicular damage associated with varicocele.[46,48]

### Abnormal blood flow

A current hypothesis assumes that increased blood flow through the testis can affect spermatogenesis.[49,50] An increase in hydrostatic pressure with a change in filtration pressure could considerably alter the composition of the interstitial fluid.[51] This alteration conceivably could alter the intimate paracrine communications between the Leydig cells, peritubular myoid cells and Sertoli cells ultimately affecting spermatogenesis.[1] The myoid cells and capillary epithelium undergo pathological changes in association with varicocele that may effect transmembrane transport of substrates to the germinal epithelium.[52]

### Endocrine imbalance

Puberty, spermatogenesis and testicular development are regulated by the hypothalamic-pituitary-testicular axis. There is a wide array of endocrine abnormalities associated with varicocele.

Leydig cells are under the control of luteinizing hormone (LH) and responsible for testosterone production. Some studies have shown that the serum testosterone level may be affected by varicocele, however it is intratesticular testosterone that is important in regulation of spermatogenesis.[53,54] In experimental animal models a varicocele can result in decreased intratesticular testosterone level.[55] The results of human studies are mixed. Ando et al. found reduced serum testosterone level in males with varicocele and increase in serum testosterone level after repair of varicocele.[56-60] Swerdloff and Walsh however showed that there was no difference in testosterone level between males with and without varicocele.[61]

Increased LH serum levels and an abnormal response to gonadotropin releasing hormone (GnRH) could implicate a compromise of the hypothalamic-pituitary-gonadal axis involved in the control of testosterone level and spermatogenesis; a pattern similar to hypergonadotropic hypogonadism.[62,63] Increased LH level results in Leydig cells hyperplasia; a known histologic finding in varicocele testicular biopsies.[64-66]

Sertoli cell responsiveness to FSH may be diminished in varicocele patients. Stimulation of Sertoli cells with FSH reversed spermatogenesis arrest in experimental animal models.[67] Altered levels of serum inhibin found in patients with varicocele may reflect altered function of Sertoli cells.[68] Cameron et al. noticed that the Sertoli-germ cell junctional complexes appeared to be structurally abnormal in patients with varicocele. They concluded that testicular disruption associated with a varicocele is a phenomenon of the adluminal compartment, and that the Sertoli cell is more sensitive to perturbation of the testicular environment than are germ cells. The Sertoli cell may be the primary intratubular site of alteration leading secondarily to spermatogenic disruption.[69] Histologic studies of the testis from patients with varicocele showed absent germ cells or altered spermatogonia to Sertoli cell ratio.[70]

### Paracrine regulations of the testis

Insight into the detailed mechanism of spermatogenesis is even more complicated as spermatogenesis is also regulated by complex interactions and signals at the cellular level in the testis.[1] (Figure 3)

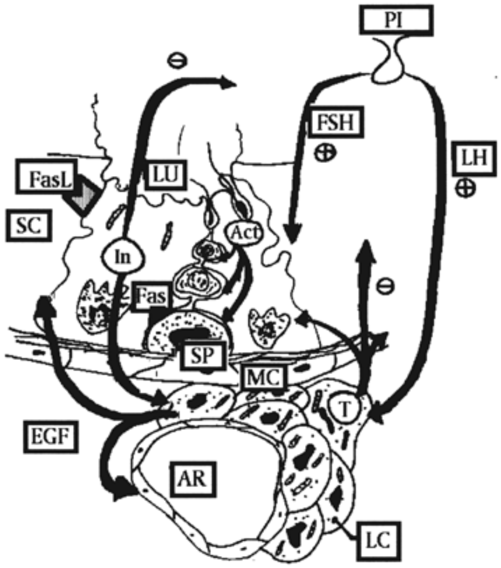

**FIGURE 3.** Endocrine regulation of spermatogenesis.

Both Sertoli cells (SC) and Leydig cells (LC) regulate spermatogenesis by steroidogenesis and growth factors production.[71,72]

Sertoli cells, tightly regulate germ cell proliferation and differentiation and are implicated in the control of germ cell apoptosis. Fas (APO-1, CD95), a transmembrane receptor protein expressed by germ cells, transmits an apoptotic signal within cells when bound by Fas ligand (FasL) produced by Sertoli cells. The Fas system has been implicated in immune regulation, including cytotoxic T cell-mediated cytotoxicity, activation-induced suicide of T cells, and control of immune-privileged sites.[73] SC stimulated by FSH produce inhibin (In) and activin (Ac).[74] Inhibin has negative feedback control on pituitary and FSH secretion. Inhibin also binds to Leydig cells (LC) regulating testosterone (T) production. Activin binds to round spermatids and spermatogonia (SP), effecting spermatogenesis. Spermatogonia are known to stimulate transferrin production by SC by an unidentified protein substance.[75]

Leydig cells control spermatogensis not only by steroids production but also by epidermal growth factors (EGF) which binds to spermatogonia and spermatids regulating cell divisions.[76] Receptors for transforming growth factor (TGF), one of the EGFs produced by LC, were found in peritubular myoid cells.[77] Peritubular myoid cells (PC) secrete peritubular myoid cell substance (PmodS) which stimulates SC. LC control adluminal tubular compartment and transport of nutrients from the vascular space to germinal epithelium by vascular endothelial growth factor (VEGF). VEGF is of particular interest in varicocele since VEGF regulates endothelial permeability and is a angioproliferative factor.[78]

Locally produced neurotropins play their distinct role in spermatogenesis regulation.[79] Opioids receptors are found on LC. During stress, release of endorphins stimulate opioid receptors and decreases testosterone production. Blocking the opioid receptors by naloxone restores testosterone production to normal.[80]

With each discovery of a new paracrine substance, and a better understanding of molecular mechanisms controlling spermatogenesis we come closer to the time when we will accurately predict which adolescent will require surgical or medical interventions for testicular dysfunction.

### PATHOLOGY

### Testicular hypotrophy

Testicular function most effected by the varicocele is spermatogenesis.[81] The most common findings on semen analysis are: increased number of pathological sperm forms, decreased motility and decreased sperm density.[8,82,83] Sperm analysis in adolescents with varicocele shows decreased sperm density, increased number of pathological forms and decreased motility however there are no established norms for adolescent semen analysis.[82] Varicocele is also associated with testicular growth arrest in adolescents.[10,12,84] Testicular growth arrest may be considered the hallmark of testicular damage in adolescent varicocele. Significant volume loss in adolescents with varicocele has been noted in 77 % of boys, 10% of whom had a left testis one-fourth the size of the right testis.[85] Testicular hypotrophy is time dependent.[86,87]

Testicular volume during preadolescents is constant and at the onset of puberty the testis suddenly increases in size even prior to other pubertal changes. In adolescents with a varicocele the rapid growth of the testis between the ages of 11 and 16 is effected by the varicocele and results in a volume discrepancy between the right and left testis.

### HISTOPATHOLOGY

Testicular biopsy in males with varicocele shows a wide array of abnormalities. The most common findings are Leydig cells hyperplasia, decreased number of spermatogonia per tubule, spermatogenesis arrest and sloughing of germinal epithelium.[88-92] A thickened basement membrane of seminiferous tubules and proliferative lesions of endoepithelium are often demonstrated and may affect transportation of oxygen and glucose through these structures.[93,94]

### DIAGNOSIS

Since the adolescent with a varicocele is often asymptomatic it is usually found on routine physical exam. The patient should be examined standing in a warm room to relax the scrotum and allow easier examination of the spermatic cord. The scrotum is first visually inspected for any obvious distention around the spermatic cord; a visible varicocele is considered a large or Grade 3 varicocele. The scrotum, testes, and cord structures are then gently palpated. A palpable varicocele has been described as feeling like a bag of worms or a squishy tube. More subtle varicoceles may feel like a thickened or asymmetric cord. The nonvisible, but palpable varicocele, is considered to be moderate in size (Grade 2). If a varicocele is not palpable but the patient performs a Valsalva maneuver which distends the pampiniform plexus of veins, then a small (Grade 1) varicocele is present. After examining the patient in the standing position, the patient should be examined supine. A thickened cord due to a varicocele should resolve in the supine position, whereas a thickened cord due to a lipoma will not change when the patient is supine. Secondary varicocele especially on the right side should always be excluded since it can be caused by serious conditions like retroperitoneal tumors, kidney tumors or lymphadenopathy.[95] Idiopathic varicocele is more prominent in the upright position and disappears in the supine position. A secondary varicocele does not change its size so dramatically in the supine position.

Testicular size needs to be measured to determine if the varicocele is adversely affecting the growth of the testis. The volume of a normal testis measures 1 to 2 ml in the prepubertal male. Due to extensive individual variation in normal growth and development, testicular size is correlated with Tanner Stage, growth velocity, and bone age rather than chronological age.[1]

A number of methods have been used to measure the size of the testis. These include visual comparison, rulers, calipers, comparative ovoids (Prader Orchidometer), punched-out elliptical rings (Takahara Orchidometer), and ultrasound. A high correlation (r = .992) between ultrasound and actual volume was noted and was shown to be highly reproducible. [96] The Prader Orchidometer was shown to correlate with ultrasound measurement in 256 patients (r = .91), though the degree of correlation was dependent upon the investigator's clinical experience. In a clinical study of 22 male adolescents with a varicocele, 24% of patients with growth arrest would have been missed if measured by Prader Orchidometer alone, and three patients felt to have a significant size discrepancy (>2 ml) by Prader Orchidometer measurements were found to be normal by ultrasound volume estimate. These findings indicate that clinical estimates of testicular size by the Prader Orchidometer are not as accurate or reproducible as those determined by ultrasound. Accurate measurement is important because operative decisions rest in the balance.[97]

There is significant disagreement as to what constitutes a significant size discrepancy justifying surgical intervention. Testicular ultrasound is the most accurate and reproducible method to assess testicular volume and significant testicular size variations. A volume difference of less than 2 ml can be due to the measurement technique alone. Therefore, size variation of greater than 2 ml by ultrasound is currently the best indicator of testicular damage and should serve as the minimal requirement for surgical repair of the adolescent varicocele. [1] Surgical intervention reverses testicular growth arrest and assessment of testicular volume postoperatively predicts resolution of the varicocele.[98]

### MANAGEMENT

There are some cardinal questions to be answered regarding management of adolescent varicocele.

Is it justified to promote the awareness about varicocele among pediatricians and primary care providers and to look for varicocele in asymptomatic adolescents?

There is sufficient evidence that varicocele is associated with testicular growth arrest in adolescents and varicocelectomy results in testis "catch-up" growth.[8,10] Lenzi et al. showed that early varicocele repair in adolescents resulted in better semen analysis results than in untreated adolescents after 2-8 years of follow up.[13] Based on these studies it seems justified to encourage non-urologists to look for varicocele in the adolescents during physical examination. Examination of the genitalia at puberty also allows the clinician to find other urologic abnormalities like cryptorchidism, hernia, curvature of penis and improve the health of adolescents.[99-102]

Once a varicocele has been found, what information needs to be given to the patient and his parents?

A number of psychological reactions (anxiety, depressed mood) were experienced in approximately 30% of boys who were informed about varicocele.[103] Since the word "infertility" is often associated with sexual impairment we believe that during discussion with the patient and his parents the only fact which should be stressed is that the varicocele may result in a decrease in testicular volume that can be reversed by surgical treatment. However it is hard to discount the association between varicocele and infertility in this era of common accessibility to the medical literature on the Internet (we found more than 50 World Wide Web pages searching for the term "varicocele", all had the word "infertility" in the text). Since there are studies which demonstrate an abnormal semen analysis in adolescents it seems advisable to discuss all the findings first with the parents who can be helpful in presentation of the problem to the child.[104,105]

Once diagnosed, who should we treat? Varicocele is the most common correctable cause of male infertility.[106] A recent metaanalysis of the literature done by Pryor and Howards showed that two thirds of patients will have improvement in semen analysis after varicocele repair, and 40 % of partners will become pregnant.[16] Historically adolescent varicocele was left untreated since its relation to infertility was not well established. Subsequently Kass and Belman showed that testicular growth arrest could be reversed by varicocelectomy in adolescents.[107] Repair of varicocele reverses not only the growth arrest but also improved semen analysis in adolescents and young males.[105,108,109]

There is good evidence that with time, if left untreated, the varicocele will continue to effect testicular growth with progressive loss of volume and progressive deterioration in semen analysis.[6, 84] Goldstein suggests that varicocele causes a progressive decline in fertility and that prior fertility in men with varicocele does not predict resistance to varicocele induced impairment of spermatogenesis in the furture so called "secondary infertility".[6]

The association between varicocele and infertility is well established.[8] The most difficult question is which clinical test should we use to establish the indications for surgical treatment in an adolescent with a varicocele.

Currently the clinical tests used to establish indications for varicocele repair are:

- Grade of varicocele
- Measurement of testicular volume to assess testicular growth arrest.
- Gonadotropin releasing hormon (GnRH) stimulation test
- Measurement of pampiniform veins diameter
- Serum luteinizing hormone (LH), follicle-stimulating hormone (FSH) and inhibin levels

### Varicocele grade

Varicocele grade does not correlate well with abnormal spermiograms or infertility in adults.[110] There are different opinions regarding correlation between grade of varicocele and degree of testicular hypotrophy in adolescents. Lyon and associates found no correlation of varicocele grade and testicular size in 30 adolescents.[85] In contrast Skoog, Steeno, and Paduch all independently noticed that boys with severe varicocele have a smaller ipsilateral testicle.[10,97,103] It was also noticed that the smaller the testis the worse the semen analysis results.[82,8] However grade of varicocele by itself should not be the sole indication for treatment.

### Testicular volume

There is an abundance of literature confirming that varicocele is associate with testicular growth arrest in adolescents and varicocele repair results in testicular "catch-up" growth.[10,105,108,109] Testicular growth arrest with volume difference of more than 2 ml assessed by ultrasonography is the most common indication for treatment.[10] The development of secondary infertility is another strong argument for early varicocele repair as if left untreated the varicocele will not only effect testicular volume but also effects spermatogenesis.[6] A decrease in testicular volume is the best indicator for surgical correction of a varicocele. However, not every boy with a varicocele and testicular growth arrest will be infertile and there is still a need to search for a test which would better distinguish between adolescents with varicocele who will develop infertility and those who will remain fertile. In adult males the situation is a little simpler because the indications for surgery are usually established after 12 months of infertility confirmed by abnormal semen analysis and the presence of a varicocele. Obtaining a semen sample in adolescents is possible but difficult.

### Gonadotropin releasing hormone (GnRH) stimulation test

Damage to germinal epithelium results in compensatory stimulation of the pituitary gland and subsequent increase in FSH and LH production by gonadotrophs. [111] Intravenous administration of GnRH stimulates the pituitary gland to release FSH and LH. FSH levels are elevated in any condition (like varicocele) effecting the integrity of germinal epithelium. [112] In theory GnRH stimulation test could distinguish between adolescents with a varicocele who have abnormal testicular functions and those who have normal spermatogenesis.[11] However clinical practice showed that the GnRH stimulating test is expensive, requires multiple serum samples and lacks the association between abnormal results, growth arrest and infertility.[113, 114] An abnormal GnRH stimulating test was found in 30 % of adolescents with varicocele and was not correlated with atrophy or infertility. [115] Currently it seems that GnRH stimulation test has limited role in clinical evaluation of adolescent with a varicocele.

### Pampiniform plexus veins diameter

Ultrasonographic measurement of pampiniform plexus veins diameter (PPVD) has been used to look for subclinical varicocele in adult infertile population when physical exam is inconclusive and to follow persistent varicocele but PPVD measurements are not useful in adolescents.[116,117]

### Inhibin level

The serum inhibin levels reflect the integrity of the seminiferous tubule. However, there is not enough data to use serum inhibin levels in clinical decisions at this time.[68,74,118-120]

Currently, prophylactic surgery for every adolescent with varicocele is not advised since it would result in treatment of 15 % of adolescents.

In summary it seems that treatment should be offered to:

- adolescents with testicular growth arrest (2 SD from normal testicular growth curves, more than 2 cc of difference between left and right testicle)
- adolescents with abnormal semen analysis with high-grade varicocele
- adolescents with symptoms: pain, heaviness, swelling
- adolescents with bilateral varicoceles

### TREATMENT OPTIONS

Treatment options in the management of adolescent with a varicocele originated from the practice of adult male infertility and subsequently were applied to the treatment of adolescents.

Varicocele repair can be done by surgical ligation and division of testicular veins or intravenous embolization of testicular veins.

Three open surgical approaches are currently used: subinguinal approach (Marmar), inguinal approach (Ivanissevich) and retroperitoneal approach (Palomo). Laparoscopic varicocele ligation has often been used in adults. Embolization techniques, regardless of embolizing material, can be divided into: antegrade (infusion through scrotal part of pampiniform plexus veins) and retrograde (catheter placed through femoral vein puncture) infusion.

The failure rate, frequency of complications, cost and outcome are important factors which need to be evaluated in choosing preferable treatment option in adolescent repair.(Table 2) It is important to remember that the majority of studies on varicocele repair relates to adult infertile males with varicocele and not adolescents.

**TABLE 2**
**Surgical approach, complications and relative costs of varicocele repair.**

| Technique | Hydrocele rate | Failure rate | Cost | References |
|---|---|---|---|---|
| Retroperitoneal | 7-10 % | 9-11 % (artery sparing)<br><3 % (artery taking) | Low | 121 10<br>12 |
| Inguinal | 3-7 % | 9-12% | Low | 123 129 147 |
| Inguinal microscopic | < 1% | 2.1 %<br>0.6% | Moderate | 124<br>125 |
| Laparoscopic | 1.25 % | 9 %<br>1.25 % in adolescents | High | 148<br>149 |
| Embolization | | 19 % | High | 135 |

### FAILURE RATE

Failure of treatment is defined here as a persistent or (rarely) recurrence of the varicocele after the repair and can occur in 9 % to 16 % of adolescents.[121] Persistence of the varicocele results in lack of "catch-up" testicular growth.[12] Most authors attribute the high recurrence rate to missed venous collaterals which run parallel to the main testicular vein. The collaterals can be quite difficult to identify and ligate separately from the testicular artery. Reported persistence rate using artery sparring retroperitoneal approach ranges from 3% to 11%.[12,121-123] Ligation of both testicular vein trunci and the artery has the advantage of a decreased persistence rate and does not result in testicular atrophy since the testis has collateral arterial blood supply from the cremasteric and deferential artery.[12,21] Atassi and colleagues achieved a persistence rate below 2 % in adolescents treated by high retroperitoneal ligation with testicular artery ligation.[12]

There is, however, some objection to simultaneous ligation of the testicular artery in men with previous inguinal surgery since there may be compromised blood supply from the cremasteric and deferential arteries. Interruption of the testicular artery in these patients has a high probability of developing testicular atrophy. Also, subsequent vasectomy in patients with testicular artery division should be avoided since ligation of the vasal artery could result in testicular atrophy in the absence of the testicular artery.

The high persistence rate and postoperative hydrocele rate resulted in the development of microsurgical inguinal approaches for correction of the varicocele. Both subinguinal and inguinal microsurgical repair are used quite often in adults with varicocele and indeed offer lower persistence rates and a low incidence of postoperative hydrocele.[124-126] The low persistence rate using the microsurgical inguinal repair is attributed to ligation of all distended vein trunci and collaterals at the level of the deep inguinal ring.[124-126] Experience in microsurgical inguinal varicocele repair in adolescent is limited. Reports by Minevitch and Goldstein demonstrated a significantly lower rate of persistence and postoperative hydrocele.[127,128] The microscopic approach allows one to ligate only the veins leaving the lymphatic vessels intact what decreased the postoperative hydrocele rate to less than 1%.[125,126]

Laparoscopic varicocele repair with, and without artery sparing modifications seems to be suitable treatment technique especially since recent reports in adults showed a lower rate of persistence.[129-131] Laparoscopic surgery in the pediatric population is gaining acceptance but it bares the risk of significant complications like bowel perforation, major vascular injury, pneumothorax, and incisional hernia. There is not much experience with laparoscopic varicocele ligation in the adolescent population.[132-134]

Retrograde embolization, unfortunately is associated with an unacceptable high rate of persistence and is the most expensive of treatment techniques.[1] Possible explanation of such a high persistence rate of the varicocele after embolization can be attributed to the highly variable anatomy of the testicular venous drainage and technical difficulties.[135] Antegrade embolization is more often used to treat persistent varicoceles than as an initial treatment.[136-139]

Other options to decrease the rate of varicocele persistence are intraoperative venography and methylene blue injections.[27,123,140,141] Intraoperative venography in theory should facilitate ligation of all testicular vein trunci and decrease the rate of persistence. Hart recommends routine use of intraoperative venography because 16% of their 62 patients had missed venous vessels after initial venous ligation.[142] Similar conclusions, based on a decreased persistence rate, were also made by Levitt, Zaontz and Gill.[143-145] However Palmer and Kass reported no difference in their rate of varicocele persistence after repair with and without intraoperative venography.[121,123,141] Based on these studies intraoperative venography offer little benefit in the repair of adolescents with a varicocele.

Based on our review of the literature and the results of a survey of pediatric urologists in the United States high retroperitoneal ligation of the testicular artery and veins offers the best results in adolescents. Although currently the high retroperitoneal approach is a treatment of choice in adolescent varicocele, pediatric urologists should consider using the microscopic inguinal or subinguinal approach with arterial preservation.

### CONCLUSIONS

The adolescent with a varicocele presents the clinician with an interesting and challenging problem. There is a great need for further basic research to help better select the patient who needs surgical correction of his varicocele.

We have outlined recommendations which can be used in everyday practice. Each clinician needs to make his/her own decisions regarding who, when and how to treat the adolescent with varicocele.

### REFERENCES

1. Skoog SJ, Roberts KP, Goldstein M, Pryor JL. The adolescent varicocele: what's new with an old problem in young patients? Pediatrics; 100 (1): 112, 1997.
2. Buch JP, Cromie WJ. Evaluation and treatment of the preadolescent varicocele. Urol Clin North Am; 12 (1): 3, 1985.
3. Vasavada S, Ross J, Nasrallah P, Kay R. Prepubertal varicoceles. Urology; 50 (5): 774, 1997.

4. Lund L, Rasmussen HH, Ernst E. Asymptomatic varicocele testis. Scand J Urol Nephrol; 27 (3): 395, 1993.
5. Niedzielski J, Paduch D, Raczynski P. Assessment of adolescent varicocele. Pediatr Surg Int; 12 (5-6): 410, 1997.
6. Gorelick JI, Goldstein M. Loss of fertility in men with varicocele. Fertil Steril; 59 (3): 613, 1993.
7. Goldstein M. New insights into the etiology and treatment of male infertility [editorial; comment]. J Urol; 158 (5): 1808, 1997.
8. The influence of varicocele on parameters of fertility in a large group of men presenting to infertility clinics. World Health Organization. Fertil Steril; 57 (6): 1289, 1992.
9. Belloli G, S DA, Pesce C, Fantuz E. [Varicocele in childhood and adolescence and other testicular anomalies: an epidemiological study]. Pediatr Med Chir; 15 (2): 159, 1993.
10. Paduch DA, Niedzielski J. Repair versus observation in adolescent varicocele: a prospective study. J Urol; 158 (3 Pt 2): 1128, 1997.
11. Kass EJ, Reitelman C. Adolescent varicocele. Urol Clin North Am; 22 (1): 151, 1995.
12. Atassi O, Kass EJ, Steinert BW. Testicular growth after successful varicocele correction in adolescents: comparison of artery sparing techniques with the Palomo procedure [see comments]. J Urol; 153 (2): 482, 1995.
13. Lenzi A, Gandini L, Bagolan P, Nahum A, Dondero F. Sperm parameters after early left varicocele treatment. Fertil Steril; 69 (2): 347, 1998.
14. Meacham RB, Townsend RR, Rademacher D, Drose JA. The incidence of varicoceles in the general population when evaluated by physical examination, gray scale sonography and color Doppler sonography. J Urol; 151 (6): 1535, 1994.
15. Di Cataldo A, Trombatore G, Di Carlo I, et al. [Idiopathic varicocele: incidence in 517 subjects]. Minerva Chir; 45 (7): 485, 1990.
16. Pryor JL, Howards SS. Varicocele. Urol Clin North Am; 14 (3): 499, 1987.
17. Opitz JM, Shapiro SS, Uehling DT. Genetic causes and workup of male and female infertility. 3. Details of the clinical evaluation. Postgrad Med; 66 (1): 129, 1979.
18. Nashan D, Behre HM, Grunert JH, Nieschlag E. Diagnostic value of scrotal sonography in infertile men: report on 658 cases. Andrologia; 22 (5): 387, 1990.
19. Jarow JP, Coburn M, Sigman M. Incidence of varicoceles in men with primary and secondary infertility. Urology; 47 (1): 73, 1996.
20. Sawczuk IS, Hensle TW, Burbige KA, Nagler HM. Varicoceles: effect on testicular volume in prepubertal and pubertal males. Urology; 41 (5): 466, 1993.
21. Parrott TS, Hewatt L. Ligation of the testicular artery and vein in adolescent varicocele. J Urol; 152 (2 Pt 2): 791, 1994.
22. Mellinger BC. Varicocelectomy. Tech Urol; 1 (4): 188, 1995.
23. Lechter A, Lopez G, Martinez C, Camacho J. Anatomy of the gonadal veins: a reappraisal. Surgery; 109 (6): 735, 1991.
24. Beck EM, Schlegel PN, Goldstein M. Intraoperative varicocele anatomy: a macroscopic and microscopic study. J Urol; 148 (4): 1190, 1992.
25. Chatel A, Bigot JM, Dectot H, Helenon C. [Radiological anatomy of the spermatic veins. Report of 152 retrograde spermatic phlebographies (author's transl)]. J Chir (Paris); 115 (8-9): 443, 1978.
26. Wishahi MM. Anatomy of the venous drainage of the human testis: testicular vein cast, microdissection and radiographic demonstration. A new anatomical concept. Eur Urol; 20 (2): 154, 1991.
27. Campobasso P. Blue venography in adolescent varicocelectomy: a modified surgical approach. J Pediatr Surg; 32 (9): 1298, 1997.
28. Wishahi MM. Detailed anatomy of the internal spermatic vein and the ovarian vein. Human cadaver study and operative spermatic venography: clinical aspects. J Urol; 145 (4): 780, 1991.
29. Wishahi MM. Anatomy of the spermatic venous plexus (pampiniform plexus) in men with and without varicocele: intraoperative venographic study. J Urol; 147 (5): 1285, 1992.
30. Shafik A, Moftah A, Olfat S, Mohi-el-Din M, el-Sayed A. Testicular veins: anatomy and role in varicocelogenesis and other pathologic conditions. Urology; 35 (2): 175, 1990.
31. Stassen CM, Weil EH, Janevski BK. Left renal vein compression syndrome ("nutcracker phenomenon"). ROFO Fortschr Geb Rontgenstr Nuklearmed; 150 (6): 708, 1989.
32. Gall H, Rudofsky G, Bahren W, Roth J, Altwein JE. [Intravascular pressure measurements and phlebography of the renal vein: a contribution to the etiology of varicocele]. Urologe [A]; 26 (6): 325, 1987.
33. Green KF, Turner TT, Howards SS. Varicocele: reversal of the testicular blood flow and temperature effects by varicocele repair. J Urol; 131 (6): 1208, 1984.
34. Nagler HM, Lizza EF, House SD, Tomashefsky P, Lipowsky HH. Testicular hemodynamic changes after the surgical creation of a varicocele in the rat. Intravital microscopic observations. J Androl; 8 (5): 292, 1987.
35. Kay R, Alexander NJ, Baugham WL. Induced varicoceles in rhesus monkeys. Fertil Steril; 31 (2): 195, 1979.
36. Fujisawa M, Yoshida S, Matsumoto O, Kojima K, Kamidono S. Deoxyribonucleic acid polymerase activity in the testes of infertile men with varicocele. Fertil Steril; 50 (5): 795, 1988.
37. Fujisawa M, Yoshida S, Matsumoto O, Kojima K, Kamidono S. Decrease of topoisomerase I activity in the testes of infertile men with varicocele. Arch Androl; 21 (1): 45, 1988.
38. Zorgniotti AW. Testis temperature, infertility, and the varicocele paradox. Urology; 16 (1): 7, 1980.

39. Hienz HA, Voggenthaler J, Weissbach L. Histological findings in testes with varicocele during childhood and their therapeutic consequences. Eur J Pediatr; 133 (2): 139, 1980.
40. Shikone T, Billig H, Hsueh A. Experimentally induced cryptorchidism increases apoptosis in rat testis. Biol Reprod; 51: 865, 1994.
41. Ibrahim AA, Hamada TA, Moussa MM. Effect of varicocele on sperm respiration and metabolism. Andrologia; 13 (3): 253, 1981.
42. Sharma RK, Agarwal A. Role of reactive oxygen species in male infertility. Urology; 48 (6): 835, 1996.
43. Mali WP, Oei HY, Arndt JW, Kremer J, Coolsaet BL, Schuur K. Hemodynamics of the varicocele. Part II. Correlation among the results of renocaval pressure measurements, varicocele scintigraphy and phlebography. J Urol; 135 (3): 489, 1986.
44. Mali WP, Arndt JW, Coolsaet BL, Kremer J, Oei HY. Haemodynamic aspects of left-sided varicocele and its association with so-called right-sided varicocele. Int J Androl; 7 (4): 297, 1984.
45. Turner TT, Lopez TJ. Effects of experimental varicocele require neither adrenal contribution nor venous reflux. J Urol; 142 (5): 1372, 1989.
46. Sofikitis N, Miyagawa I. Left adrenalectomy in varicocelized rats does not inhibit the development of varicocele-related physiologic alterations. Int J Fertil Menopausal Stud; 38 (4): 250, 1993.
47. York JP, Klump R, Smith JJ, Drago JR. The role of the adrenal in the rat varicocele model. In Vivo; 4 (2): 145, 1990.
48. Steeno O, Koumans J, De Moor P. Adrenal cortical hormones in the spermatic vein of 95 patients with left varicocele. Andrologia; 8 (2): 101, 1976.
49. Harrison RM, Lewis RW, Roberts JA. Testicular blood flow and fluid dynamics in monkeys with surgically induced varicoceles. J Androl; 4 (4): 256, 1983.
50. Saypol DC, Howards SS, Turner TT, Miller ED, Jr. Influence of surgically induced varicocele on testicular blood flow, temperature, and histology in adult rats and dogs. J Clin Invest; 68 (1): 39, 1981.
51. Sweeney TE, Rozum JS, Gore RW. Alteration of testicular microvascular pressures during venous pressure elevation. Am J Physiol; 269 (1 Pt 2): H37, 1995.
52. Santamaria L, Martin R, Nistal M, Paniagua R. The peritubular myoid cells in the testes from men with varicocele: an ultrastructural, immunohistochemical and quantitative study. Histopathology; 21 (5): 423, 1992.
53. Su LM, Goldstein M, Schlegel PN. The effect of varicocelectomy on serum testosterone levels in infertile men with varicoceles. J Urol; 154 (5): 1752, 1995.
54. Hampl R, Lachman M, Novak Z, Sulcova J, Starka L. Serum levels of steroid hormones in men with varicocele and oligospermia as compared to normozoospermic men. Exp Clin Endocrinol; 100 (3): 117, 1992.
55. Rajfer J, Turner TT, Rivera F, Howards SS, Sikka SC. Inhibition of testicular testosterone biosynthesis following experimental varicocele in rats. Biol Reprod; 36 (4): 933, 1987.
56. Ando S, Giacchetto C, Beraldi E, et al. Testosterone and dihydrotestosterone seminal plasma levels in varicocele patients. Acta Eur Fertil; 13 (3): 113, 1982.
57. Ando A, Giacchetto C, Beraldi E, et al. The influence of age on Leydig cell function in patients with varicocele. Int J Androl; 7 (2): 104, 1984.
58. Ando S, Giacchetto C, Colpi G, et al. Physiopathologic aspects of Leydig cell function in varicocele patients. J Androl; 5 (3): 163, 1984.
59. Ando S, Giacchetto C, Beraldi E, Panno ML, Carpino A, Brancati C. Progesterone, 17-OH-progesterone, androstenedione and testosterone plasma levels in spermatic venous blood of normal men and varicocele patients. Horm Metab Res; 17 (2): 99, 1985.
60. Ando S, Giacchetto C, Colpi GM, Beraldi E, Panno ML, Sposato G. Testosterone precursors in spermatic venous blood of normal men and varicocele patients. A study of delta 4 pathway of testosterone biosynthesis. Acta Endocrinol (Copenh); 108 (2): 277, 1985.
61. Swerdloff RS, Walsh PC. Pituitary and gonadal hormones in patients with varicocele. Fertil Steril; 26 (10): 1006, 1975.
62. Kass EJ, Freitas JE, Bour JB. Adolescent varicocele: objective indications for treatment. J Urol; 142 (2 Pt 2): 579, 1989.
63. Bickel A, Dickstein G. Factors predicting the outcome of varicocele repair for subfertility: the value of the luteinizing hormone-releasing hormone test. J Urol; 142 (5): 1230, 1989.
64. McFadden MR, Mehan DJ. Testicular biopsies in 101 cases of varicocele. J Urol; 119 (3): 372, 1978.
65. Hadziselimovic F, Leibundgut B, Da Rugna D, Buser MW. The value of testicular biopsy in patients with varicocele. J Urol; 135 (4): 707, 1986.
66. Sirvent JJ, Bernat R, Navarro MA, Rodriguez Tolra J, Guspi R, Bosch R. Leydig cell in idiopathic varicocele. Eur Urol; 17 (3): 257, 1990.
67. Sofikitis N, Takahashi C, Kadowaki H, et al. Surgical repair versus medical treatment of varicocele in the rat: pharmacological manipulation of the varicocelized testicle. Eur Urol; 22 (1): 44, 1992.
68. Plymate SR, Paulsen CA, McLachlan RI. Relationship of serum inhibin levels to serum follicle stimulating hormone and sperm production in normal men and men with varicoceles [published erratum appears in J Clin Endocrinol Metab 1992 Oct;75(4):1059]. J Clin Endocrinol Metab; 74 (4): 859, 1992.

69. Cameron DF, Snydle FE, Ross MH, Drylie DM. Ultrastructural alterations in the adluminal testicular compartment in men with varicocele. Fertil Steril; 33 (5): 526, 1980.
70. Cameron DF, Snydle FE. Ultrastructural surface characteristics of seminiferous tubules from men with varicocele. Andrologia; 14 (5): 425, 1982.
71. Schlatt S, Meinhardt A, Nieschlag E. Paracrine regulation of cellular interactions in the testis: factors in search of a function. European Journal of Endocrinology; 137 (2): 107, 1997.
72. Schlatt S, Arslan M, Weinbauer GF, Behre HM, Nieschlag E. Endocrine control of testicular somatic and premeiotic germ cell development in the immature testis of the primate Macaca mulatta. European Journal of Endocrinology; 133 (2): 235, 1995.
73. Lee J, Richburg JH, Younkin SC, Boekelheide K. The Fas system is a key regulator of germ cell apoptosis in the testis. Endocrinology; 138 (5): 2081, 1997.
74. Mather JP, Moore A, Li RH. Activins, inhibins, and follistatins: further thoughts on a growing family of regulators. Proceedings of the Society for Experimental Biology & Medicine; 215 (3): 209, 1997.
75. Boujrad N, Hochereau-de Reviers MT, Carreau S. Evidence for germ cell control of Sertoli cell function in three models of germ cell depletion in adult rat. Biology of Reproduction; 53 (6): 1345, 1995.
76. Yan YC, Sun YP, Zhang ML. Testis epidermal growth factor and spermatogenesis. Archives of Andrology; 40 (2): 133, 1998.
77. Nakazumi H, Sasano H, Maehara I, Orikasa S. Transforming growth factor-alpha, epidermal growth factor, and epidermal growth factor receptor in human testis obtained from biopsy and castration: immunohistochemical study. Tohoku Journal of Experimental Medicine; 178 (4): 381, 1996.
78. Ergun S, Kilic N, Fiedler W, Mukhopadhyay AK. Vascular endothelial growth factor and its receptors in normal human testicular tissue. Molecular & Cellular Endocrinology; 131 (1): 9, 1997.
79. Seidl K, Buchberger A, Erck C. Expression of nerve growth factor and neurotrophin receptors in testicular cells suggest novel roles for neurotrophins outside the nervous system. Reproduction, Fertility, & Development; 8 (7): 1075, 1996.
80. Kostic T, Andric S, Kovacevic R, Maric D. The effect of opioid antagonists in local regulation of testicular response to acute stress in adult rats. Steroids; 62 (11): 703, 1997.
81. Micic S, Illic V, Isvaneski M. Correlation of hormone and histologic parameters in infertile men with varicocele. Urol Int; 38 (3): 187, 1983.
82. Paduch DA, Niedzielski J. Semen analysis in young men with varicocele: preliminary study. J Urol; 156 (2 Pt 2): 788, 1996.
83. Nagao RR, Plymate SR, Berger RE, Perin EB, Paulsen CA. Comparison of gonadal function between fertile and infertile men with varicoceles. Fertil Steril; 46 (5): 930, 1986.
84. Sayfan J, Siplovich L, Koltun L, Benyamin N. Varicocele treatment in pubertal boys prevents testicular growth arrest. J Urol; 157 (4): 1456, 1997.
85. Lyon RP, Marshall S, Scott MP. Varicocele in childhood and adolescence: implication in adulthood infertility? Urology; 19 (6): 641, 1982.
86. Lipshultz LI, Corriere JN, Jr. Progressive testicular atrophy in the varicocele patient. J Urol; 117 (2): 175, 1977.
87. Witt MA, Lipshultz LI. Varicocele: a progressive or static lesion? Urology; 42 (5): 541, 1993.
88. Aragona F, Ragazzi R, Pozzan GB, et al. Correlation of testicular volume, histology and LHRH test in adolescents with idiopathic varicocele. Eur Urol; 26 (1): 61, 1994.
89. Ponchietti R, Grechi G, Dini G. Varicocele in adolescents: ultrastructural aspects. Acta Eur Fertil; 17 (1): 47, 1986.
90. Kass EJ, Chandra RS, Belman AB. Testicular histology in the adolescent with a varicocele. Pediatrics; 79 (6): 996, 1987.
91. Hadziselimovic F, Herzog B, Jenny P. The chance for fertility in adolescent boys after corrective surgery for varicocele. J Urol; 154 (2 Pt 2): 731, 1995.
92. Castro-Magana M, Angulo M, Canas A, Uy J. Leydig cell function in adolescent boys with varicoceles. Arch Androl; 24 (1): 73, 1990.
93. Hadziselimovic F, Herzog B, Liebundgut B, Jenny P, Buser M. Testicular and vascular changes in children and adults with varicocele. J Urol; 142 (2 Pt 2): 583, 1989.
94. Chakraborty J, Hikim AP, Jhunjhunwala JS. Stagnation of blood in the microcirculatory vessels in the testes of men with varicocele. J Androl; 6 (2): 117, 1985.
95. Abdominal mass with varicocele. N Y State J Med; 78 (14): 2219, 1978.
96. Behre HM, Nashan D, Nieschlag E. Objective measurement of testicular volume by ultrasonography. Int J Androl; 12: 395, 1989.
97. Costabile RA, Skoog S, Radowich M. Testicular volume assessment in the adolescent with a varicocele. J Urol; 147 (5): 1348, 1992.
98. Gentile DP, Cockett AT. The effect of varicocelectomy on testicular volume in 89 infertile adult males with varicoceles. Fertil Steril; 58 (1): 209, 1992.
99. Nagar H, Levran R. Impact of active case-finding on the diagnosis and therapy of pediatric varicocele. Surg Gynecol Obstet; 177 (1): 38, 1993.

100. Oster J. Clinical phenomena noted by a school physician dealing with healthy children. Clin Pediatr (Phila); 15 (8): 748, 1976.
101. Oster J. Varicocele in children and adolescents. Scand J Urol Nephrol; 5: 27, 1971.
102. Gres AA, Shmygira MB. [Urologic diseases in boys and adolescents found during targeted prophylactic examinations]. Urol Nefrol (Mosk) (4-6): 40, 1992.
103. Steeno O, Knops J, Declerck L, Adimoelja A, van de Voorde H. Prevention of fertility disorders by detection and treatment of varicocele at school and college age. Andrologia; 8 (1): 47, 1976.
104. Yamamoto M, Hibi H, Katsuno S, Miyake K. Effects of varicocelectomy on testis volume and semen parameters in adolescents: a randomized prospective study. Nagoya J Med Sci; 58 (3-4): 127, 1995.
105. Laven JS, Haans LC, Mali WP, te Velde ER, Wensing CJ, Eimers JM. Effects of varicocele treatment in adolescents: a randomized study. Fertil Steril; 58 (4): 756, 1992.
106. Greenberg SH, Lipshultz LI, Wein AJ. Experience with 425 subfertile male patients. J Urol; 119 (4): 507, 1978.
107. Kass EJ, Belman AB. Reversal of testicular growth failure by varicocele ligation. J Urol; 137 (3): 475, 1987.
108. Okuyama A, Nakamura M, Namiki M, et al. Surgical repair of varicocele at puberty: preventive treatment for fertility improvement [see comments]. J Urol; 139 (3): 562, 1988.
109. Haans LC, Laven JS, Mali WP, te Velde ER, Wensing CJ. Testis volumes, semen quality, and hormonal patterns in adolescents with and without a varicocele. Fertil Steril; 56 (4): 731, 1991.
110. Vereecken RL, Boeckx G. Does fertility improvement after varicocele treatment justify preventive treatment at puberty? Urology; 28 (2): 122, 1986.
111. Hudson RW, McKay DE. The gonadotropin response of men with varicoceles to gonadotropin-releasing hormone. Fertil Steril; 33 (4): 427, 1980.
112. Hudson RW, Crawford VA, McKay DE. The gonadotropin response of men with varicoceles to a four-hour infusion of gonadotropin-releasing hormone. Fertil Steril; 36 (5): 633, 1981.
113. Haidl G, Maass C, Schill WB. When to treat varicocele? Acta Chir Hung; 34 (3-4): 309, 1994.
114. Hudson RW. The endocrinology of varicoceles. Fertil Steril; 49 (2): 199, 1988.
115. Kass EJ, Freitas JE, Salisz JA, Steinert BW. Pituitary gonadal dysfunction in adolescents with varicocele [see comments]. Urology; 42 (2): 179, 1993.
116. Winkelbauer F, Karnel F, Ammann ME, Hofbauer J. [Ultrasound diagnosis of persistent varicocele after sclerotherapy]. Ultraschall Med; 15 (1): 29, 1994.
117. Aydos K, Baltaci S, Salih M, Anafarta K, Beduk Y, Gulsoy U. Use of color Doppler sonography in the evaluation of varicoceles. Eur Urol; 24 (2): 221, 1993.
118. Pryor JP, Pugh RC, Cameron KM, Newton JR, Collins WP. Plasma gonadotrophic hormones, testicular biopsy and seminal analysis in the men of infertile marriages. Br J Urol; 48 (7): 709, 1976.
119. Baccetti B, Burrini AG, Capitani S, et al. Studies on varicocele. 1. Submicroscopical and endocrinological features. J Submicrosc Cytol Pathol; 23 (4): 659, 1991.
120. Baccetti B, Burrini AG, Capitani S, et al. Studies on varicocele. II. The inhibin secretion. J Submicrosc Cytol Pathol; 25 (1): 137, 1993.
121. Kass EJ, Marcol B. Results of varicocele surgery in adolescents: a comparison of techniques. J Urol; 148 (2 Pt 2): 694, 1992.
122. Allouch G. [Varicocele in adolescents. 67 cases]. J Urol (Paris); 102 (2): 62, 1996.
123. Palmer LS, Maizels M, Kaplan WE, Stokes S, Firlit CF. The influence of surgical approach and intraoperative venography on successful varicocelectomy in adolescents. J Urol; 158 (3 Pt 2): 1201, 1997.
124. Marmar JL, Kim Y. Subinguinal microsurgical varicocelectomy: a technical critique and statistical analysis of semen and pregnancy data. J Urol; 152 (4): 1127, 1994.
125. Goldstein M, Gilbert BR, Dicker AP, Dwosh J, Gnecco C. Microsurgical inguinal varicocelectomy with delivery of the testis: an artery and lymphatic sparing technique. J Urol; 148 (6): 1808, 1992.
126. Chalouhy E, Kassardjian Z, Merhej S, et al. Microsurgical high inguinal varicocelectomy with delivery of the testis. J Med Liban; 42 (3): 105, 1994.
127. Minevich E, Wacksman J, Lewis AG, Sheldon CA. Inguinal microsurgical varicocelectomy in the adolescent: technique and preliminary results. J Urol; 159 (3): 1022, 1998.
128. Lima M, Domini M, Libri M. The varicocele in pediatric age: 207 cases treated with microsurgical technique. Eur J Pediatr Surg; 7 (1): 30, 1997.
129. Ulker V, Garibyan H, Kurth KH. Comparison of inguinal and laparoscopic approaches in the treatment of varicocele. Int Urol Nephrol; 29 (1): 71, 1997.
130. al-Shareef ZH, Koneru SR, al-Tayeb A, Shehata ZM, Aly TF, Basyouni A. Laparoscopic ligation of varicoceles: an anatomically superior operation [see comments]. Ann R Coll Surg Engl; 75 (5): 345, 1993.
131. Wuernschimmel E, Lipsky H, Noest G. Laparoscopic varicocele ligation: a recommendable standard procedure with good long-term results. Eur Urol; 27 (1): 18, 1995.
132. Seibold J, Janetschek G, Bartsch G. Laparoscopic surgery in pediatric urology. Eur Urol; 30 (3): 394, 1996.
133. Fahlenkamp D, Winfield HN, Schonberger B, Mueller W, Loening SA. Role of laparoscopic surgery in pediatric urology. Eur Urol; 32 (1): 75, 1997.

134. Humphrey GM, Najmaldin AS. Laparoscopy in the management of pediatric varicoceles. J Pediatr Surg; 32 (10): 1470, 1997.
135. Feneley MR, Pal MK, Nockler IB, Hendry WF. Retrograde embolization and causes of failure in the primary treatment of varicocele. Br J Urol; 80 (4): 642, 1997.
136. Johnsen N, Johnsen I, Tauber R. Semen analysis after treatment of varicocele by antegrade scrotal sclerotherapy. Adv Exp Med Biol; 424: 187, 1997.
137. Kuenkel MR, Korth K. Rationale for antegrade sclerotherapy in varicoceles. Eur Urol; 27 (1): 13, 1995.
138. Johnsen N, Tauber R. Financial analysis of antegrade scrotal sclerotherapy for men with varicoceles. Br J Urol; 77 (1): 129, 1996.
139. Mottrie AM, Matani Y, Baert J, Voges GE, Hohenfellner R. Antegrade scrotal sclerotherapy for the treatment of varicocele in childhood and adolescence. Br J Urol; 76 (1): 21, 1995.
140. Belloli G, S DA, Musi L, Campobasso P. Adolescent varicocele: operative anatomy and tricks for successful correction. Eur J Pediatr Surg; 5 (4): 219, 1995.
141. Palmer LS, Cohen S, Reda EF, et al. Intraoperative spermatic venography reconsidered. J Urol; 154 (1): 225, 1995.
142. Hart RR, Rushton HG, Belman AB. Intraoperative spermatic venography during varicocele surgery in adolescents. J Urol; 148 (5): 1514, 1992.
143. Levitt S, Gill B, Katlowitz N, Kogan SJ, Reda E. Routine intraoperative post-ligation venography in the treatment of the pediatric varicocele. J Urol; 137 (4): 716, 1987.
144. Zaontz MR, Firlit CF. Use of venography as an aid in varicocelectomy. J Urol; 138 (4 Pt 2): 1041, 1987.
145. Gill B, Kogan SJ, Maldonado J, Reda E, Levitt SB. Significance of intraoperative venographic patterns on the postoperative recurrence and surgical incision placement of pediatric varicoceles. J Urol; 144 (2 Pt 2): 502, 1990.
146. Yerokhin A. Classification and frequency of varicocele in children. Klin Khir; 6: 45, 1979.
147. Dubin L, Amelar RD. Varicocelectomy: twenty-five years of experience. Int J Fertil; 33 (4): 226, 1988.
148. Dahlstrand C, Thune A, Hedelin H, Grenabo L, Pettersson S. Laparoscopic ligature of the spermatic veins. A comparison between outpatient and hospitalised treatment. Scand J Urol Nephrol; 28 (2): 159, 1994.
149. Belloli G, Musi L, S DA. Laparoscopic surgery for adolescent varicocele: preliminary report on 80 patients. J Pediatr Surg; 31 (11): 1488, 1996.

**This article should be referenced as follows:**

Paduch, D.A. and Skoog, S.J. (2004) Diagnosis, evaluation and treatment of adolescent varicocele. *TheScientificWorldJOURNAL* **4 (S1)**, 263–278.

**Handling Editor:**

Anthony Atala, Principle Editor for *Urology* — a domain of *TheScientificWorldJOURNAL*.
